# Mangrove growth and biomass dynamics along the mud-dominated coast of French Guiana

**DOI:** 10.1038/s41598-026-53756-1

**Published:** 2026-05-21

**Authors:** Michael Kyei Agyekum, Joao Marcelo Brazao Protazio, Adrien Staquet, Paul-Emile Augusseau, Antoine Gardel, Antoine Mury, Edward J Anthony, Christophe Proisy

**Affiliations:** 1 Helmholtz Centre for Environmental Research, Magdeburg, Germany; 2 Faculty 2 Biology/Chemistry, University of Bremen, Bremen, Germany; 3 Federal University of Para (UFPA), Belem, Brazil; 4 AMAP, IRD, CIRAD, CNRS, INRAE, Univ. Montpellier, Montpellier, France; 5 Centre National de la Recherche Scientifique, LEEISA, Cayenne, France; 6 iSEA, Pessac, France; 7 Aix Marseille University, CNRS, IRD, INRAE, Coll France, CEREGE, Aix-en-Provence, France; 8 ICUB, University of Bucharest, Bucharest, 050663 Romania

**Keywords:** Growth models, Aboveground biomass, Accretion, Mudbanks, Erosion, Chronosequence, Ecology, Ecology, Environmental sciences

## Abstract

Mangrove forests fringe the highly dynamic, mud-dominated Guianas coast, where wave-induced mobility of Amazon-derived mudbanks drives rapid coastal accretion and erosion. In French Guiana, this geomorphic instability reorganizes mangrove distribution, creating mosaics of successional stages in which stand development rarely approaches long-term equilibrium. However, it remains uncertain whether a single age–biomass relationship can represent contrasting coastal Avicennia-dominated stands and heterogeneous estuarine Rhizophora-dominated stands in this system. This study evaluates how effectively stand age, reconstructed from historical imagery, predicts stem diameter at breast height (DBH) and aboveground biomass (AGB) of *Avicennia germinans* and *Rhizophora* spp. along this coast, using chronosequence data spanning pioneer to mature stands. DBH was measured in the field, and AGB was estimated using locally validated allometric equations. Four empirical growth models (power, Gompertz, logistic, and monomolecular) were fitted using nonlinear least squares to describe age–structure and age–biomass relationships. Stand age strongly predicted DBH in *Avicennia germinans* but explained less variation in AGB and in both DBH and AGB for *Rhizophora* spp., and differences among growth functions were small. In this mud‑dominated setting, stand‑age models perform well for coastal mangroves but are less informative for estuarine mangroves. Under ongoing climate and environment‑driven change, this study highlights the need to complement stand age with environmental and structural covariates when modelling mangrove biomass and carbon stocks in both coastal and estuarine systems.

**Supplementary Information:**

The online version contains supplementary material available at 10.1038/s41598-026-53756-1.

## Introduction

**Fig. 1:**
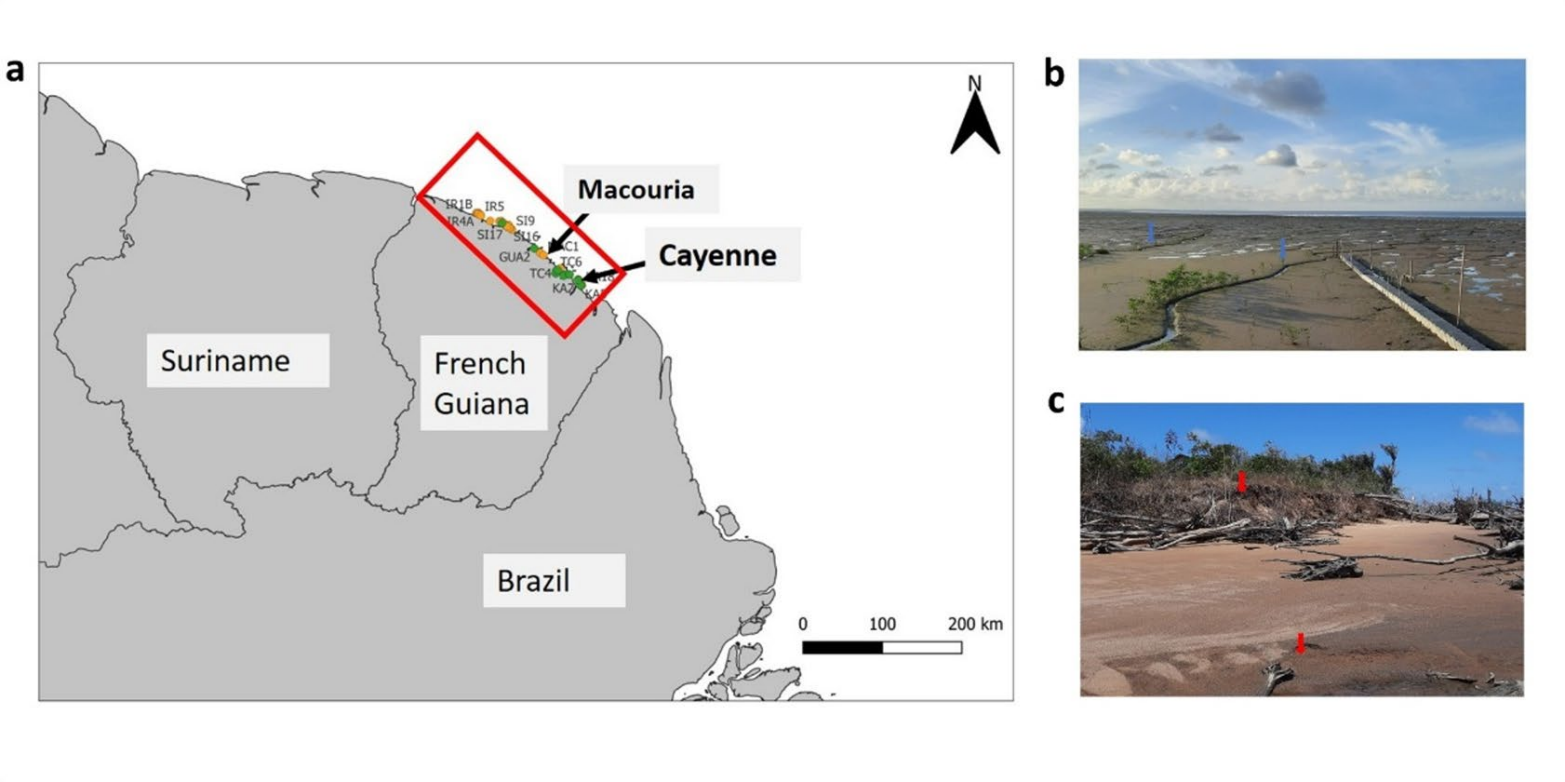
The mud-dominated coast of French Guiana and ground photographs showing active accretion and erosion. **a** Location of the study area and sampling plots along the French Guiana mangrove coast, indicated by the red box. Orange points indicate coastal *Avicennia*-dominated plots and green points indicate estuarine *Rhizophora*-dominated plots. The labelled locations indicate the areas represented by the example photographs in panels **b** and **c**. **b** Active accretion near Cayenne, with blue arrows showing sediment deposition and pioneer colonisation. **c** Coastal erosion near Macouria, with red arrows indicating the erosion scarp and mangrove die-back. The map was produced in QGIS version 3.38.3 (QGIS Development Team; https://www.qgis.org) using the French Guiana subnational administrative boundaries and coastline from the COD-AB GUF dataset provided by IGN through the Humanitarian Data Exchange (https://data.humdata.org/dataset/cod-ab-guf).

Mangrove forests along the Guianas (Fig. 1) coast develop within a highly dynamic, mud-dominated shoreline system, where extensive Amazon derived mudbanks drive pronounced coastal instability^1–5^. In French Guiana, these processes create and erase habitat, so that mangrove forests are continuously reorganized in space and time, with recurrent cycles of forest establishment, development, and erosion-driven loss^6–9^. These contrasting phases of coastal accretion, forest development and erosion are illustrated for the study region in Fig. 1.

In such sediment-dominated coastal systems, mangrove forests are rarely in long-term equilibrium^10^. Instead, forest structure primarily reflects the time elapsed since substrate emergence and colonization, superimposed on local environmental conditions^11,12^. Previous work in French Guiana has shown that mangrove stands form a mosaic of age classes linked to mudbank dynamics, enabling chronosequence analyses of stand development even in the absence of reliable dendrochronological markers^10,13^. In these coastal systems, stand age has therefore emerged as a key organising variable for understanding mangrove structure, aboveground biomass and carbon stocks, particularly where long‑term plot data are scarce and age must be reconstructed from historical imagery^10,14,15^.

In contrast, estuarine mangroves develop more independently of coastal mudbank dynamics, and stand age is not directly constrained by shoreline evolution; in such settings, age reconstruction would require alternative approaches, such as growth‑ring analysis at the tree level, which has been demonstrated for *Rhizophora* in North Brazil^16^.

Mangrove growth dynamics are often quantified using empirical growth models that relate forest stand-level attributes, such as stem diameter or aboveground biomass, to stand age^17,18^. In forest ecology, a range of empirical growth functions is used to describe stand development, including asymptotic and sigmoid forms such as Gompertz, logistic and monomolecular models, as well as non-asymptotic power functions used in specific contexts^19–22^. In mangrove research, these models have been applied to describe stem growth, biomass accumulation and carbon sequestration across chronosequences, but their performance can be influenced by uncertainty in stand age and species-specific growth strategies^2,10,17,23,24^. Mangrove taxa differ markedly in architecture, wood density, and growth allocation, which influence biomass accumulation patterns and can affect both the form and robustness of age-based growth relationships^18,25^.

Along the Guianas coast, *Avicennia germinans* commonly dominates extensive, even aged coastal stands on newly accreted substrates, whereas *Rhizophora* spp. are more frequently associated with structurally complex estuarine and interior settings where hydrological gradients are stronger^2,10,26,27^. Previous work along the exposed mudbank coast has shown that coastal *Avicennia* stands follow relatively coherent structural trajectories linked to mudbank migration and shoreline change, so that stand age and coastal dynamics can be meaningfully related at the landscape scale^2,10,26^. In contrast, estuarine and interior *Rhizophora* mosaics can include co‑occurring dwarf and very tall tree formations within a few kilometres, indicating that stand structure diverges strongly even for similar apparent ages^10,25^.

Most chronosequence based studies implicitly assume that stand age provides a reliable proxy for cumulative biomass accumulation^28,29^ such that forests of similar age follow broadly comparable biomass trajectories despite local variability^10,30^. This assumption underlies the widespread use of age-based models to estimate mangrove biomass and carbon stocks where long-term monitoring data are lacking. Along the mud-dominated coast of French Guiana, however, strong geomorphic instability, hydrological heterogeneity and species-specific growth strategies may decouple time since establishment from stand structure and biomass development. As a result, the validity of age–biomass relationships across contrasting coastal and estuarine stands in this system remains uncertain.

In this study, we analysed stand age-related growth dynamics of *Avicennia germinans* and *Rhizophora* spp. along the mud-dominated coast of French Guiana using stand ages reconstructed from historical imagery and four commonly applied empirical growth functions. We examined how strongly stand age predicts variation in stem diameter and aboveground biomass, whether age is more informative for diameter than for biomass, and whether alternative growth models differ in their performance. By comparing these taxa within a geomorphically unstable sedimentary environment, this study clarifies both the potential and the limitations of age-based approaches for modelling structure, biomass and carbon stocks in coastal and estuarine dominated mangrove settings.

## Results

Descriptive statistics indicate clear differences in stand-level structural attributes between the two taxa. Values are reported as mean ± standard deviation (SD), with medians and ranges. For *Avicennia germinans* (*n* = 46), mean diameter at breast height (DBH) was 32.70 ± 20.07 cm (median = 25.16 cm), with values ranging from 7.71 to 87.85 cm. Mean aboveground biomass (AGB) was 146.26 ± 74.39 t DM ha⁻¹ (median = 151.09 t DM ha⁻¹), spanning 4.77 to 304.47 t DM ha⁻¹. For *Rhizophora* spp. (*n* = 21), mean DBH was 10.10 ± 7.73 cm (median = 7.16 cm), ranging from 1.77 to 29.14 cm. Mean AGB was 10.70 ± 26.49 t DM ha⁻¹ (median = 1.41 t DM ha⁻¹), ranging from 0.004 to 120.82 t DM ha⁻¹, reflecting the presence of many low-biomass stands and a small number of high-biomass stands.

### Growth dynamics of Avicennia germinans DBH and biomass

**Fig. 2:**
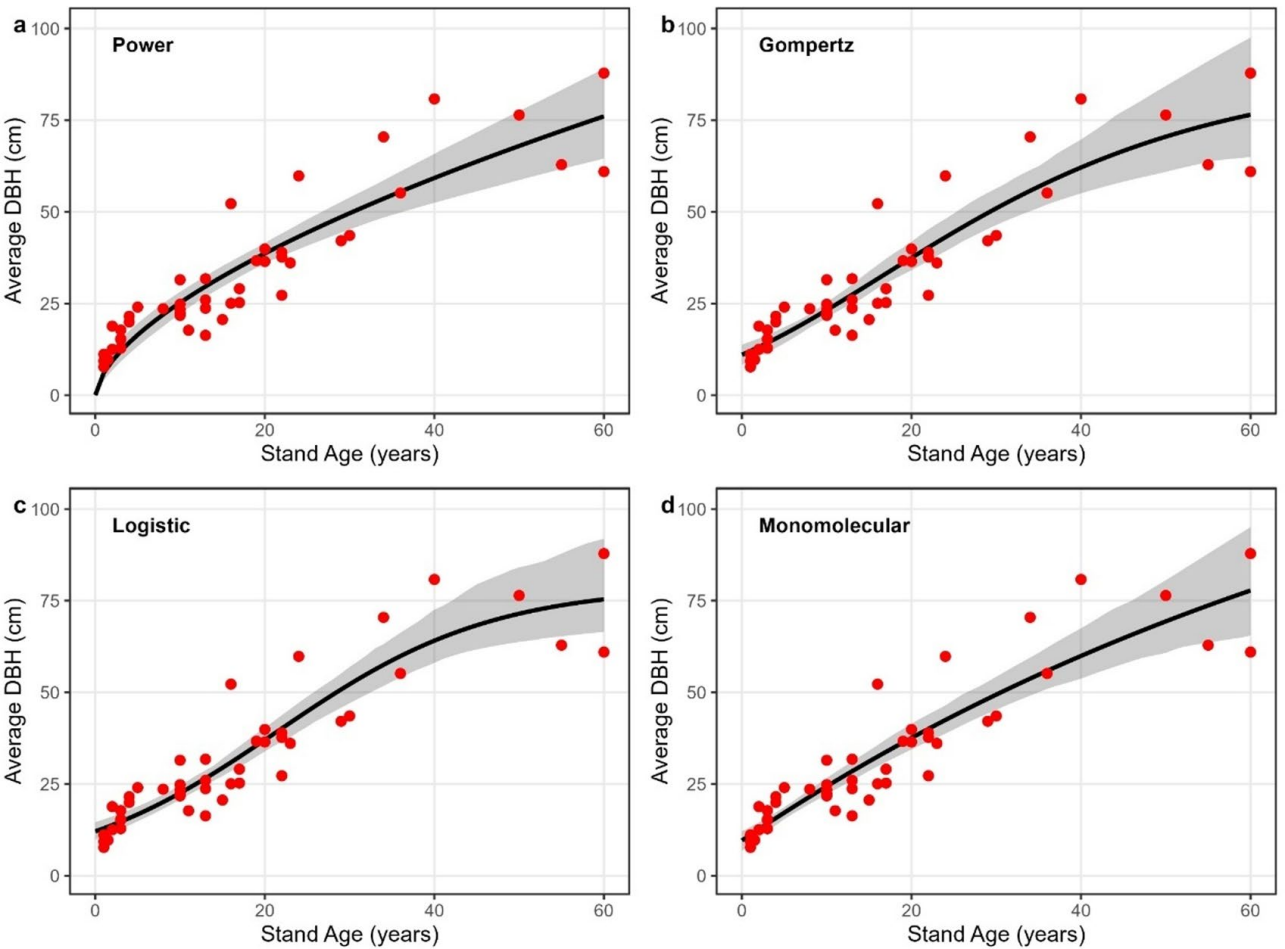
Stand age–DBH relationships for *Avicennia germinans*. Observed stand-level mean DBH (points) and fitted growth curves (lines) for **a** Power, **b** Gompertz, **c** Logistic **d** Monomolecular. Shaded bands represent 95% bootstrap uncertainty bands around the fitted mean growth curve, obtained by refitting models to resampled datasets. Stand age is in years and DBH in centimeters. The plots were created using the ggplot2 package in R.

Stand age–diameter at breast height (DBH) relationships for *Avicennia germinans* showed a consistent increase in diameter with age, followed by a gradual leveling at older stand ages (Fig. 2). Across the four growth models, pseudo R² values ranged from 0.82 to 0.85, RMSE from 6.84 to 8.43 cm, and MAE from 5.86 to 6.62 cm, with AIC values between 324.03 and 332.24 (Table 1). The logistic model yielded the highest pseudo R² (0.85), whereas the Gompertz model showed the lowest AIC (324.03). However, ΔAIC values across models were generally modest, indicating that all functional forms provided broadly similar descriptions of the stand age-DBH relationship.

**Table 1:** Goodness-of-fit statistics for nonlinear growth models fitted to stand-level diameter at breast height (DBH) and aboveground biomass (AGB) of *Avicennia germinans*. Four model forms (Power, Gompertz, Logistic, and Monomolecular) were evaluated. Reported metrics include a pseudo R² (calculated as the squared Pearson correlation between observed and fitted values), Akaike Information Criterion (AIC), ΔAIC relative to the best-supported model within each response variable, root mean square error (RMSE), and mean absolute error (MAE).

Species	Parameter	Model	*R* ^2^	AIC	ΔAIC	RMSE	MAE
***Avicennia germinans***	DBH	Power	0.82	330.71	6.68	8.43	6.62
		Gompertz	0.84	324.03	0.0	6.84	5.92
		Logistic	0.85	332.24	8.21	7.69	5.86
		Monomolecular	0.84	326.51	2.48	8.06	5.92
	AGB	Power	0.52	496.09	0.58	50.89	40.10
		Gompertz	0.52	495.86	0.35	50.77	41.16
		Logistic	0.52	496.10	0.59	50.91	41.38
		Monomolecular	0.53	495.51	0.00	50.58	40.76

**Fig. 3:**
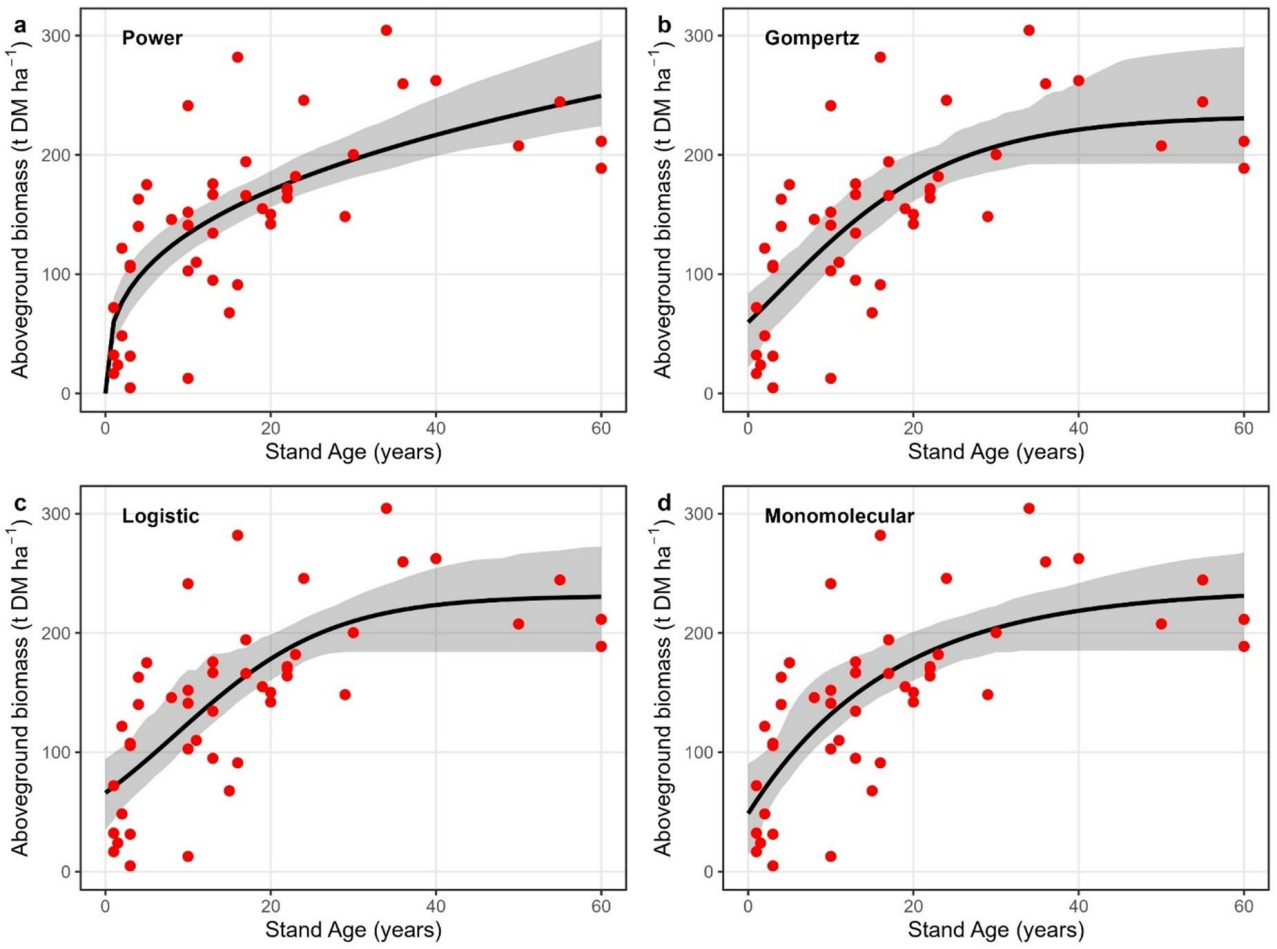
Stand age–AGB relationships for *Avicennia germinans*. Observed stand-level aboveground biomass (points) and fitted growth curves (lines) for **a** Power, **b** Gompertz, **c** Logistic **d** Monomolecular. Shaded bands represent 95% bootstrap uncertainty bands around the fitted mean growth curve, obtained by refitting models to resampled datasets. Stand age is in years and AGB in t DM ha⁻¹. The plots were created using the ggplot2 package in R.

Stand age-AGB relationships for *Avicennia germinans* also showed increasing biomass with age but with substantially higher variability than observed for DBH (Fig. 3). The fitted curves indicated rapid biomass accumulation at younger to intermediate stand ages, followed by a tendency toward levelling at older ages, particularly in the Gompertz, logistic and monomolecular models. For AGB, pseudo R² values ranged from 0.52 to 0.53, RMSE from 50.58 to 50.91 t DM ha⁻¹, and MAE from 40.10 to 41.38 t DM ha⁻¹, with AIC values between 495.51 and 496.10 (Table 1). The monomolecular model achieved the highest pseudo R² (0.53) and the lowest AIC (495.51), but ΔAIC across all models remained less than or equal to 5, indicating no clear support for a single functional form.

### Growth dynamics of Rhizophora spp. DBH and biomass

**Fig. 4:**
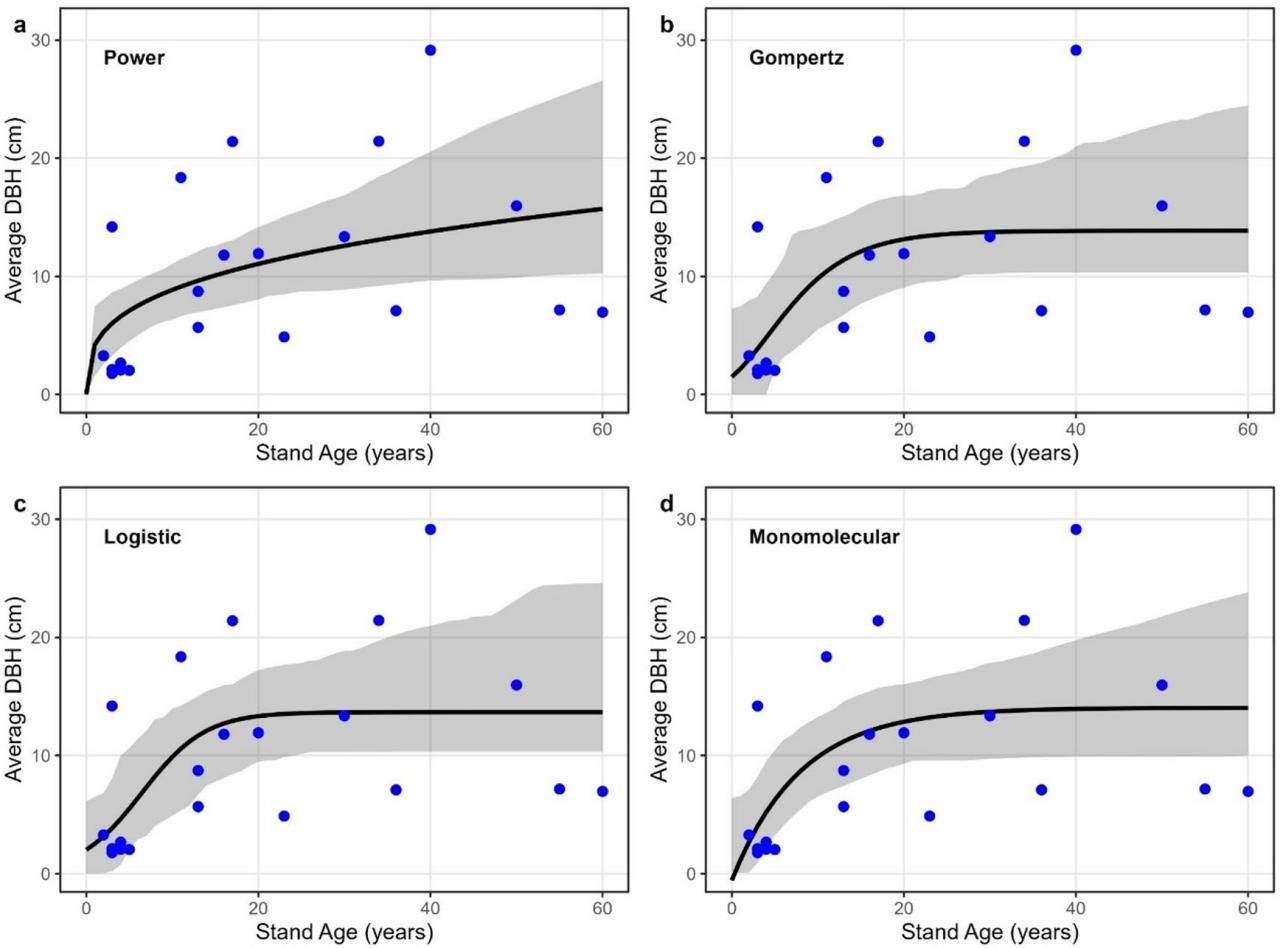
Stand age–DBH relationships for *Rhizophora* spp. Observed stand-level mean DBH (points) and fitted growth curves (lines) for **a** Power, **b** Gompertz, **c** Logistic **d** Monomolecular. Shaded bands represent 95% bootstrap uncertainty bands around the fitted mean growth curve, obtained by refitting models to resampled datasets. The plots were created using the ggplot2 package in R.

For *Rhizophora* spp., DBH increased with stand age but exhibited greater scatter than observed for *Avicennia germinans* (Fig. 4). Pseudo R² values ranged from 0.23 (power) to 0.31 (Gompertz and logistic), RMSE from 6.28 to 6.62 cm, and MAE from 4.96 to 5.41 cm, with AIC values between 144.75 and 145.03 (Table 2). Gompertz and logistic models yielded the highest pseudo R² (0.31) and the lowest AIC values, but ΔAIC across all models was less than or equal to 0.3, indicating weak differentiation among functional forms and limited explanatory power of stand age alone.

**Table 2:** Goodness-of-fit statistics for nonlinear growth models fitted to stand-level diameter at breast height (DBH) and aboveground biomass (AGB) of *Rhizophora* spp. Four model forms (Power, Gompertz, Logistic, and Monomolecular) were evaluated. Reported metrics include a pseudo R² (calculated as the squared Pearson correlation between observed and fitted values), Akaike Information Criterion (AIC), ΔAIC relative to the best-supported model within each response variable, root mean square error (RMSE), and mean absolute error (MAE).

Species	Parameter	Model	*R* ^2^	AIC	ΔAIC	RMSE	MAE
***Rhizophora*** **spp.**	DBH	Power	0.23	145.03	0.28	6.62	5.41
		Gompertz	0.31	144.82	0.07	6.29	4.97
		Logistic	0.31	144.75	0.00	6.28	4.96
		Monomolecular	0.30	144.97	0.22	6.31	5.01
	AGB	Power	0.34	139.06	0.00	8.25	5.48
		Gompertz	0.32	140.24	1.18	8.60	5.30
		Logistic	0.32	139.76	0.70	8.90	5.14
		Monomolecular	0.28	140.67	1.61	8.39	5.75

**Fig. 5:**
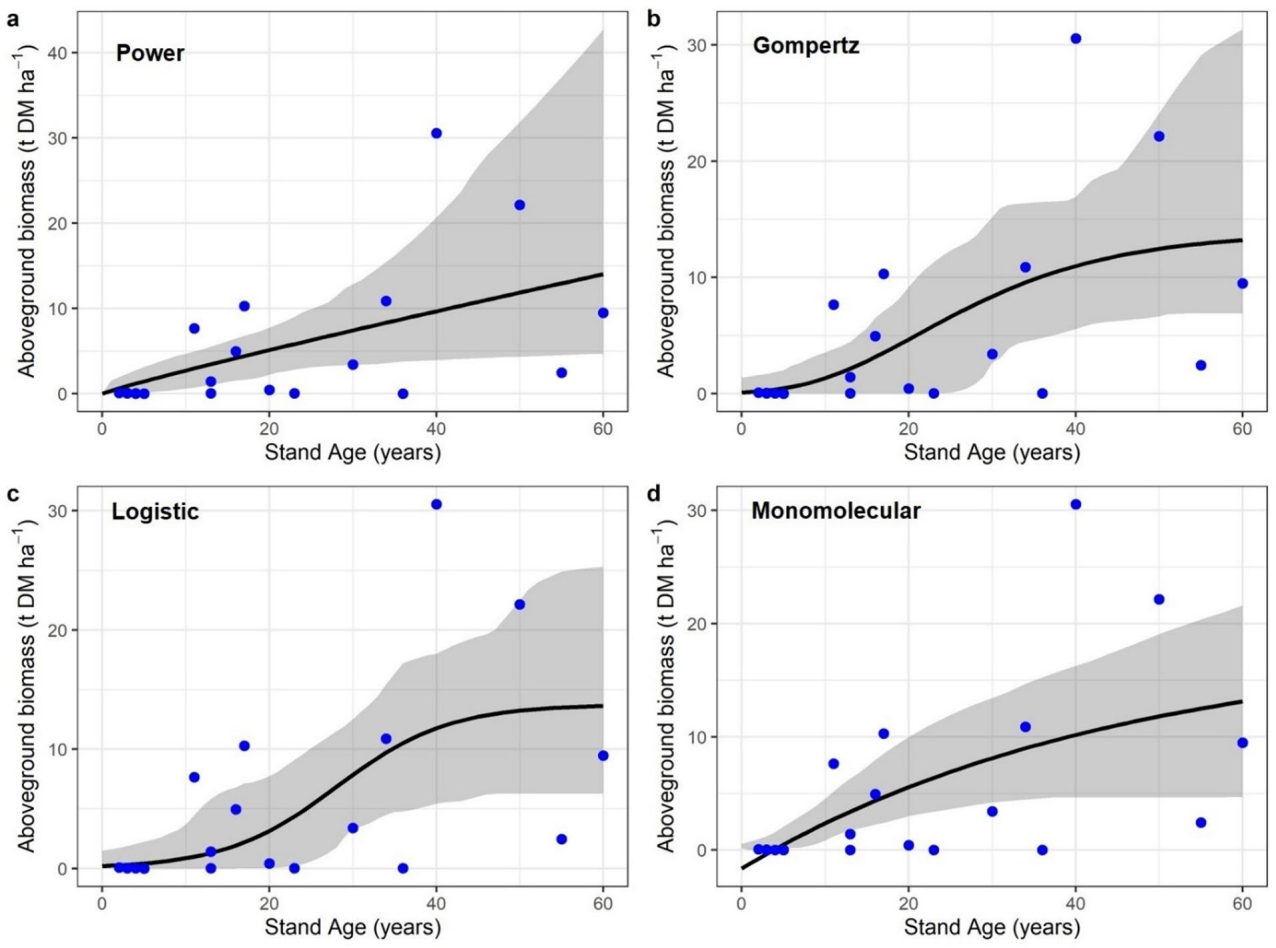
Stand age–AGB relationships for *Rhizophora* spp. Observed stand-level aboveground biomass (points) and fitted growth curves (lines) for **a** Power, **b** Gompertz, **c** Logistic **d** Monomolecular. Shaded bands represent 95% bootstrap uncertainty bands around the fitted mean growth curve, obtained by refitting models to resampled datasets. Stand age is in years and AGB in t DM ha⁻¹. The plots were created using the ggplot2 package in R.

Age to AGB relationships for *Rhizophora* spp. showed the lowest overall model performance (Fig. 5). Across models, pseudo R² values ranged from 0.28 to 0.34, RMSE from 8.25 to 8.90.

t DM ha⁻¹, and MAE from 5.14 to 5.75 t DM ha⁻¹, with AIC values between 139.06 and 140.67 (Table 2). Although the power model produced the highest pseudo R² (0.34), ΔAIC differences among models were small, indicating that biomass to age relationships for *Rhizophora* spp. were only weakly explained by stand age irrespective of model choice.

Stand structural variables were further examined in relation to stand age and residual variation from the age-based models (Figs. S2–S5). In the plot-level data, stand basal area generally increased with stand age in both taxa, whereas stem-density patterns differed between taxa. For Avicennia germinans, AGB residuals showed a weak positive trend with basal area and little consistent trend with stem density (Fig. S2). For Rhizophora spp., AGB residuals showed a stronger positive trend with basal area and a positive but variable trend with stem density (Fig. S3). DBH residuals showed positive trends with basal area in both taxa, while relationships with stem density differed between taxa, being weakly negative for Avicennia germinans and weakly positive for Rhizophora spp. (Figs. S4, S5).

## Discussion

This study shows that chronosequence‑based growth models can capture stand‑scale diameter development in *Avicennia germinans* along the highly dynamic, mud‑dominated coast of French Guiana, while revealing important limits of age‑based approaches for predicting biomass and stand structure in more heterogeneous estuarine *Rhizophora* settings. In practical terms, this means that stand‑age models perform well for coastal mangroves established on newly accreted mudbanks, but are much less informative for long‑lived, estuarine *Rhizophora* stands that develop more independently of shoreline dynamics.

The contrasting responses of *Avicennia germinans* and Rhizophora spp. reflect their different geomorphic and ecological settings along the Guianas coast. Repeated cycles of mudbank accretion and erosion create a mosaic of mangrove stands of different ages on newly formed substrates^31^. Within this setting, *Avicennia germinans* often form extensive, relatively even-aged stands on accreting mudbanks, whereas *Rhizophora* spp. occur more frequently along riverine, estuarine, and interior gradients^2^. As a result, the development of *Rhizophora* stands is less directly constrained by coastal mudbank dynamics than that of Avicennia‑dominated coastal forests, which helps to explain the weaker age–structure and age–biomass signals observed for *Rhizophora* in this study. These patterns are consistent with earlier work in French Guiana, where strong spatial variability in mangrove structure, biomass, and stand development has been linked to sediment dynamics and successional stage^13^.

The age–DBH relationships observed for *Avicennia germinans* indicate that stand age captures a substantial component of structural variation at the stand scale, consistent with previous reports of well‑defined structural chronosequences in *Avicennia*‑dominated mangroves in the Guianas region and elsewhere^10^. In this context, the chronosequence framework quantifies the variability of structural outcomes among stands of similar apparent age rather than assuming a single uniform trajectory. Even in Avicennia-dominated stands where stand age strongly predicts mean DBH, residual variation reflects differences in competitive structure and space occupancy that are not captured by age alone. This may suggest that age-based chronosequences describe average developmental trajectories, while individual stands deviate according to local stand structure.

For Rhizophora spp., the weaker associations between age and DBH (maximum pseudo R² of 0.31) and the higher variability around fitted curves indicate that, in this dataset, stand age alone represents only a limited part of the variation in stand structure. This pattern is consistent with the ecological distribution of Rhizophora along pronounced hydrological and salinity gradients and with studies reporting higher structural variability in stands of similar apparent age in estuarine and riverine settings^32^. In such environments, differences in hydroperiod, salinity, nutrient availability, surface elevation, and sedimentary setting may lead to divergent growth trajectories among stands of comparable age^11,32^. Age-based biomass accumulation has also been modelled in mangrove systems outside French Guiana, including Rhizophora plantations in Vietnam^30^, while recent work in Rhizophora-dominated mangroves on the Great Barrier Reef showed that aboveground biomass was related to both forest age and tidal position^15^. Previous mangrove carbon studies further show that hydrogeomorphic setting, land-use history, and coastal environmental setting can strongly influence mangrove carbon storage and dynamics^25^. In addition, the complex architecture of Rhizophora, including aerial root systems and multi-stemmed or highly branched forms, may weaken the correspondence between mean trunk DBH, stand age, and stand-level biomass^18,26^. These relationships should therefore be interpreted as statistical associations, with stand age acting as a proxy for cumulative exposure to site conditions rather than as evidence that age alone determines stand structure^28^. Any species-specific differences within Rhizophora spp. are therefore subsumed within the observed variability.

The weaker age–AGB relationships compared with age–DBH relationships highlight the greater structural complexity of biomass accumulation. Across both taxa, stand age was more strongly associated with DBH than with AGB, consistent with the different ways these variables integrate stand development: mean DBH reflects cumulative radial growth of stems, whereas stand‑level biomass integrates tree size distributions and stem density via allometric equations. In *Avicennia germinans*, the combination of relatively high pseudo R^2^ for DBH, moderate pseudo R^2^ for AGB, and wide biomass variation among stands of similar age suggests that variation in stand density, size structure, or disturbance history contributes substantially to stand‑level biomass but is not captured by age alone.

Although plot sizes varied substantially, this primarily affects sampling variability rather than systematically biasing area-standardised stand-level estimates. The weak age–AGB relationships persisted across plot sizes and stand ages, indicating that the limited explanatory power of age was not driven by plot area. Pseudo R^2^ values of 0.28 to 0.34 for *Rhizophora* AGB are modest. However, they are consistent with the moderate explanatory power typically observed for biomass–age relationships in mangrove chronosequences, particularly where stand age integrates heterogeneous geomorphic and hydrological conditions and only a limited set of predictors is available. For Rhizophora spp., the modest AGB model performance is also consistent with previous reports of high between-stand variability in Rhizophora biomass for a given age class^10,23,26,32^. Together, these patterns indicate that age-based models provide, at best, a first-order approximation of stand-level biomass in French Guiana^10^, particularly for *Rhizophora* and for structurally heterogeneous stands. High variability in very young stands reflects rapid post-colonisation restructuring and does not bias conclusions regarding long-term age dependence.

Differences among the four growth functions were small relative to the unexplained variation in biomass, especially for Rhizophora spp. The four growth functions examined are among the most widely used empirical models in forest ecology. For *Avicennia germinans* DBH, all models achieved similar goodness-of-fit, indicating that when the age–structure signal is strong, alternative sigmoidal and monotone formulations can describe the data comparably well. For stand-level biomass, and especially for *Rhizophora* spp., differences among models were small relative to the unexplained variance, suggesting that variation may arise predominantly from ecological and structural factors not represented in the age-based models. This is consistent with findings from other forest systems where changes in growth function have limited effect on predictive performance when key environmental or stand-level covariates are absent^17,30^. Given the limited sample size for *Rhizophora* and the relatively simple three-parameter structure of the models, model form alone does not explain the small ΔAIC values. Instead, these values are more consistent with the limited information content of age as a sole predictor in this case.

The space-for-time interpretation is most robust for Avicennia-dominated coastal stands and less certain for heterogeneous Rhizophora stands. Space-for-time approaches are widely used in mangrove ecology to infer temporal dynamics from spatial patterns, particularly where long-term growth records are lacking^10^.

The strong age–DBH relationships for *Avicennia germinans* in this study support the use of space-for-time to describe diameter development in *Avicennia*‑dominated stands on newly accreted substrates along the French Guiana coast^2,4,26^. However, the weaker age–AGB relationships and the low explanatory power of age for *Rhizophora* biomass highlight important limitations when space-for-time assumptions are extended to biomass estimation or to taxa occupying more heterogeneous hydrological and geomorphic settings^2,10^. In such cases, chronosequence models that include additional covariates such as geomorphic setting, hydroperiod, salinity, or stand density are likely to provide more robust inferences than age-based formulations^25^. Under ongoing climate‑ and sea‑level change, coastal settings differing in sediment supply, wave climate, and relative sea‑level trends are expected to follow distinct growth trajectories, so that age–structure relationships calibrated for French Guiana should not be assumed to apply uniformly to other mangrove shorelines^2,24,31,33^.

Chronosequence-based inference remains approximate in a coastal system shaped by repeated disturbance and shoreline reorganisation. The chronosequence interpretation relies on the assumption that differences among plots within a given geomorphic setting primarily reflect differences in time since establishment, rather than distinct disturbance histories^34^. In a system as dynamic as the French Guiana coast, where repeated erosion, accretion, and shoreline reorganisation restructure mangrove stands over decadal timescales^2,6^, this assumption is necessarily approximate. It is therefore likely to be more robust for extensive, relatively even-aged coastal Avicennia stands, where establishment is closely tied to mudbank accretion^2,10^, than for *Rhizophora* stands developing within more heterogeneous estuarine and interior settings where hydrogeomorphic gradients and disturbance histories vary strongly^25^.

The results have direct implications for biomass and carbon-stock assessment in dynamic mangrove systems. Accurate stand-level biomass estimates are central to assessments of mangrove carbon stocks and fluxes and increasingly rely on combinations of field data, allometric equations, and spatially explicit predictors such as age or canopy structure derived from remote sensing. The moderate to weak performance of age-based biomass models observed here indicates that stand age, taken alone, is insufficient for management-relevant biomass estimation, particularly in structurally complex estuarine *Rhizophora* stands. Age maps may nevertheless support first-order biomass estimates in extensive, relatively even-aged *Avicennia germinans* stands, provided that associated uncertainties are explicitly quantified and, where possible, reduced by including structural metrics (e.g. canopy height, stand density) or geomorphic classes as additional predictors. For both coastal Avicennia‑dominated mangroves and estuarine *Rhizophora*‑dominated forests, incorporating environmental and structural factors into growth models will therefore be essential to capture changing trajectories under future climate and coastal forcing.

Two methodological aspects are particularly relevant for interpreting these findings. First, stand ages were reconstructed from historical imagery in discrete intervals, which introduces uncertainty that may attenuate age–response relationships, especially for older stands. The age-jitter sensitivity analysis indicated that plausible age perturbations altered pseudo R² and AIC only modestly for *Avicennia germinans* DBH, but could contribute substantially to the residual variance observed for biomass and *Rhizophora* metrics, reinforcing the interpretation that age-based models provide coarse constraints on these responses. Second, biomass estimates were derived from allometric equations applied to stand-level DBH data, which, although validated for the study region, add uncertainty that cannot be fully resolved by empirical growth modelling. Future work could address these limitations by explicitly propagating age uncertainty in growth models, integrating environmental and structural covariates obtained from field measurements or remote sensing, and combining chronosequence analyses with repeated plot measurements to test the robustness of age-based inferences in dynamic coastal and estuarine mangrove systems.

In conclusion, stand age reconstructed from historical imagery provides a useful first-order predictor of diameter development in Avicennia-dominated coastal mangroves of French Guiana, but it is less reliable for predicting aboveground biomass and for describing structurally heterogeneous Rhizophora stands. The results show that age-based chronosequence models are most informative where stand establishment is closely linked to coastal mudbank accretion, and less informative where local hydrological, geomorphic and structural variability is high. Incorporating structural attributes, environmental covariates and remotely sensed canopy information will therefore be important for improving mangrove biomass and carbon-stock estimates in dynamic coastal and estuarine systems.

## Materials and methods

### Study Area

Mangrove forests in French Guiana extend along approximately 320 km of the Atlantic seaboard, covering about 70,000 ha within the Amazon sediment dispersal system^2^. The northeastern coast of South America receives large inputs of fine sediments sourced from the Amazon Basin, which are transported northwestward alongshore as part of the Amazon dispersal system in both suspended load and as migrating mudbanks several tens of kilometers long^2^. These shoreface-attached mudbanks are major drivers of coastal morphodynamics along the Guianas coast, alternating between phases of mudbank welding and inter-bank erosion that can lead to accretion of new sedimentary surfaces or to shoreline retreat during erosion-dominant periods^33^. This alternation of phases creates a shifting mosaic of geomorphological conditions that promotes opportunistic mangrove colonization on newly accreted sediments and forest establishment, followed by periods of erosion that can remove mature mangrove stands (Fig. 1).

The mangrove community is floristically simple, dominated by three taxa. *Avicennia germinans* form expansive, tall, often even aged stands and are the most widespread species. *Rhizophora* spp. (*R. mangle and R. racemosa*) are most abundant along riverine and estuarine gradients where salinity is moderated by freshwater input. *Laguncularia racemosa*, a heliophilous species, specializes in the colonization of newly stabilized banks. Zonation of these taxa reflects key ecological filters: *Avicennia germinans* tolerate high salinity and unstable mudbanks, *Rhizophora* spp. generally occupy more stable inland formations, and *Laguncularia racemosa* dominates disturbed, high-light seafront sites. This combination of high physical dynamism and species-specific strategies makes French Guiana an ideal setting to test competing models of mangrove growth. All study-area maps and plot-location maps were produced in QGIS version 3.38.3 (QGIS Development Team) using the French Guiana subnational administrative boundaries and coastline from the COD-AB GUF dataset provided by IGN through the Humanitarian Data Exchange.

### Field Sampling

Fieldwork encompassed the full successional gradient, from pioneer stands to late stage forests. In total, 69 plots were surveyed along the French Guiana coast, including stands dominated by *Avicennia germinans* and *Rhizophora* spp. Two plots were excluded from age-based modeling due to ambiguous canopy establishment date, resulting in effective sample sizes of 46 (*Avicennia germinans*) and 21 (*Rhizophora* spp.) for stands less than 70 years after excluding mixed or high-uncertainty stands.

Plot areas ranged from 7 to 10,000 m² because sampling area was adjusted to stand structure, the spatial extent of homogeneous vegetation patches, and field accessibility. Smaller plots were used in young, dense, or spatially very homogeneous pioneer stands, particularly on newly accreted mudbanks where larger plots were not feasible. Larger plots were used in older or more heterogeneous stands to capture representative stand conditions. Although smaller plots may exhibit greater sampling variability due to lower stem numbers, the use of stand-level mean DBH and area-standardised biomass ensures that plot size does not systematically bias stand-level estimates of DBH or AGB.

For each plot, stem density (tree stems ha⁻¹) was determined as the number of tree stems per plot area, and stand basal area (m² ha⁻¹) was estimated from DBH measurements and plot area (see Eqs. 1–3). These metrics were used descriptively to characterise stand structure and to aid interpretation of biomass variability, but were not included as covariates in the age-based growth models. Sampling was stratified across stands representing a range of apparent ages and geomorphic contexts associated with mudbank accretion and subsequent forest development. Within each geomorphic context, plots were selected to minimise obvious confounding disturbances such as recent clearing or partial die-back. Although the sampling design captures the main successional stages and geomorphic settings, some bias towards more accessible coastal stands cannot be excluded.

Species dominance within each plot was defined using basal area contribution (Table S1). A plot was classified as *Avicennia germinans* dominated or *Rhizophora* spp. dominated when the focal taxon contributed at least 70% of total basal area, a threshold commonly used to define functional dominance in mixed mangrove stands. Plots not meeting this criterion were classified as mixed stands and were excluded from species-specific growth model fitting.

Within each plot, all living stems with diameter at breast height (DBH; 1.3 m) ≥ 4 cm were measured. Aboveground biomass (AGB) was estimated using published allometric equations validated for *Avicennia germinans*^13^(Eq. 4) and *Rhizophora* spp.^10^. (Eq. 5) in French Guiana. These equations were selected because they were developed and validated for the same taxa, size ranges, and environmental conditions, and their parameter values fall within the range reported for mangrove allometries in comparable neotropical systems. Individual tree biomass was calculated using species-specific allometries, summed per plot, and standardized by plot area (Eq. 6) to obtain stand-level AGB (t DM ha⁻¹). Because the growth analyses were conducted using stand-level response variables, uncertainty in observed DBH and AGB was summarized as variability among stand-level plot records. Variation in tree size within plots was incorporated into the calculation of stand-level DBH and AGB, but within-plot confidence intervals were not estimated separately.

The basal area for an individual tree Gi and for the stand Gtot as well as the average DBH per mangrove stand (where N is the number of trees per hectare) are given by:

Basal area

1 $$\:Gi\left({m}^{2}\right)=\frac{\pi\:.{DBHi}^{2}\:}{4x{10}^{4}}$$

2 $$\:Gtot\left({m}^{2}h{a}^{-1}\right)=\sum\:\frac{Gi\:}{Area}*{10}^{4}$$

3 $$\:Average\:DBH\:\left(cm\right)=200*\sqrt{\frac{Gtot}{\pi\:N}}$$

4 $$\:AGB{A}_{A\left(g\right)}=160*DB{H}^{2.42}$$

5 $$\:AGB{A}_{R\left(g\right)}=128*DB{H}^{2.61}$$

Total stand-level aboveground biomass was expressed as tonnes of dry matter per hectare.

(t DM ha⁻¹) using:

6 $$\:AGBtot\:\left(\:t\:DM\:h{a}^{-1}\right)=\sum\:\frac{AGBix{10}^{4}}{Area\:x\:{10}^{6}}$$

where AGB_i_ is individual tree biomass in grams, Area is plot area in m², 10^4^ converts m² to hectares, and 10^6^ converts grams to tonnes.

### Stand age reconstruction

Stand age (years since establishment) was derived from georeferenced historical satellite and aerial imagery (1940–2022; IGN *Remonter le temps*). Details of the imagery sources used for stand-age reconstruction, including platform type, sensor or image type, approximate period used, typical spatial resolution, and their role in identifying persistent woody canopy, are provided in Supplementary Table S2. For each stand, the first appearance of persistent woody canopy was identified across sequential images. Plot coordinates were overlaid on georeferenced imagery in ArcMap 10.8.2. Historical aerial photographs and orthophotographs were accessed via the IGN *Remonter le temps* portal, which provides georeferenced and, where available, orthorectified imagery derived from national aerial surveys. Satellite imagery, including SPOT and Landsat data, was used in its standard georeferenced and provider-corrected form. Spatial alignment among imagery sources was visually checked using stable landscape features such as shorelines and river channels prior to interpretation. Canopy establishment was then determined by visual interpretation of the first occurrence of persistent woody canopy that remained visible in subsequent images.

This imagery‑based stand‑age reconstruction was applied consistently to both coastal Avicennia‑dominated and estuarine *Rhizophora*‑dominated stands. Representative historical imagery illustrating shoreline progradation and canopy establishment is provided in Fig. S1. Age uncertainty reflects the temporal resolution of available imagery, which typically ranges from about 5 to 10 years between acquisitions for most of the study period. Although stand age was reconstructed within discrete imagery intervals, the uncertainty classes were applied consistently across plots and taxa according to image availability. Within the primary modelled dataset, age uncertainty was therefore treated as an imagery-derived source of uncertainty rather than as a species-specific effect. Estimated stand ages are reported in the Supplementary material (see Table S2)^35^.

Analyses rely on a space-for-time substitution, assuming that stands of different ages represent stages along a common successional trajectory under broadly comparable environmental conditions. To assess the influence of age uncertainty on model performance, a sensitivity analysis was conducted in which stand ages were randomly perturbed within their imagery-derived intervals and growth models were refitted to each perturbed dataset (Tables S2 and S3). This sensitivity analysis was designed to assess the robustness of fitted growth trajectories to plausible age uncertainty, rather than to formally propagate age uncertainty into parameter inference or model selection.

### Growth models

Stand-level growth dynamics were modelled using empirical growth equations widely applied in forest ecology and mangrove research to describe age-related changes in structural attributes^17,21^. Four commonly used nonlinear growth models relating stand age to mean DBH or aboveground biomass were fitted: two sigmoidal models (Gompertz and logistic), a monomolecular model (monotone asymptotic), and a power function (non-asymptotic). Together, these models span a range of functional forms from unbounded growth to asymptotic saturation and allow comparison of alternative representations of age–size relationships^17,23^.

The selected equations differ in their assumptions about early growth rates, curvature, and the presence or absence of an asymptotic upper limit. Sigmoidal models such as the Gompertz (Eq. 7) and logistic (Eq. 8) functions have been widely used to describe diameter and biomass development in mangrove forests, as they capture rapid early growth followed by progressive deceleration as stands mature^17,23,36^. The monomolecular model (Eq. 9) allows more gradual saturation dynamics and has been applied in cases where asymptotic behaviour is less pronounced or varies among stands^37^. The power model (Eq. 10), which does not impose an asymptote, was included to test whether the empirical data support unbounded age–response relationships, a relevant consideration in highly dynamic mangrove systems subject to recurrent disturbance and sediment-driven reorganisation^33,35^. Analyses were restricted to stands less than 70 years old to prioritize the portion of the chronosequence with the lowest age uncertainty (± 5 years, due to dense post-1980 imagery coverage with 3–5 year intervals). Mature stands (nominal age = 70 years, ± 10 years uncertainty from sparse mid-century imagery gaps of 8–12 years) were excluded from primary model fitting. Sensitivity checks confirmed that including these high-uncertainty mature stands substantially degraded model performance, with pseudo R² decreasing by 0.07–0.17 units and RMSE increasing by 1.4–10× across DBH and aboveground biomass variables for both *Avicennia germinans* and *Rhizophora* spp.

All models were fitted in their original formulations to stand age–DBH and stand age–aboveground biomass data using identical fitting and evaluation procedures^2,17,26,38^.

Gompertz model:

7 $$\:y\left(t\right)={\theta\:}_{1}{e}^{-{\theta\:}_{2}{e}^{-{\theta\:}_{3}t}}$$

Logistic model:

8 $$\:{y\left(t\right)=\theta\:}_{1}/[1+{\theta\:}_{2}{e}^{-{\theta\:}_{3}t}]$$

Monomolecular model:

9 $$\:y\left(t\right)={\theta\:}_{1}[1-{\theta\:}_{2}{e}^{-{\theta\:}_{3}t}]$$

Power model:

10 $$\:y\left(t\right)={\theta\:}_{1}{t}^{{\theta\:}_{2}}$$

Here *y* is DBH (cm) or AGB (t DM ha⁻¹), t is stand age (years), θ₁ is the upper asymptote (for asymptotic models), θ₂ is a rate or scale parameter, and θ₃ is a shape parameter where applicable. Initial parameter values and bounds were constrained to biologically plausible ranges (e.g., θ₁>0, θ₂>0, θ₃>0) and were selected within intervals reported in previous mangrove and forest growth studies for comparable species and stand structures^17,37–39^. Where necessary, these literature-based ranges were slightly adjusted to ensure numerical convergence for the French Guiana dataset (Table S3) while remaining ecologically realistic^35^.

### Model fitting and evaluation

Models were fitted using nonlinear least squares regression^37^. Starting values for parameters were derived from simple exploratory fits and from published parameterizations of the same or closely related growth models for mangroves and other tropical forests, and were checked to ensure that fitted trajectories remained within ecologically realistic bounds for DBH and AGB^35^.

Stand diameter (DBH) and above-ground biomass (AGB) were modelled as functions of stand age using a suite of nonlinear growth functions, including Power, Gompertz, Logistic, and Monomolecular formulations, fitted separately for *Avicennia germinans* and *Rhizophora* spp. *Rhizophora* stands were analysed at the genus level because field plots frequently contained mixed individuals of *R. racemosa* and *R. mangle*, and species-level age reconstruction from imagery was not feasible. Observed DBH and AGB values represent stand-level means derived from plot measurements and were used as the response variables in all growth model fits. The analysis focused on stand age as a single explanatory variable to evaluate the limits of chronosequence-based inference in highly dynamic coastal settings. Including environmental covariates would confound this objective by introducing site-specific drivers that are not consistently reconstructable over multi-decadal time frames. Model comparison was used to assess the robustness of age-based relationships rather than to identify a single ‘best’ growth function. To evaluate the sensitivity of fitted growth curves to sampling variability, a nonparametric bootstrap with 200 resamples was applied. Models were repeatedly refitted to resampled datasets, and fitted mean responses were predicted across stand age to derive age-specific bootstrap uncertainty bands. Bootstrap results were used solely for graphical assessment of uncertainty around the fitted curves and were not used for parameter inference or model selection.

All nonlinear regressions were implemented in the R environment using the *nls* function with the bounded *port* algorithm to constrain parameters to biologically realistic ranges. Residuals were visually inspected against stand age and fitted values, and no strong systematic patterns were observed. Model performance was assessed using pseudo R², root mean square error (RMSE), mean absolute error (MAE), and Akaike Information Criterion (AIC), calculated from residuals of the original fitted models. All goodness-of-fit metrics were computed consistently across species and models using residuals from the original nonlinear fits. For each species–parameter combination, models were ranked using ΔAIC, defined as the difference between a given model’s AIC and the minimum AIC among the candidate set. ΔAIC values were used to quantify the relative support for each model following an information-theoretic approach. To further assess whether stand structural attributes contribute to variability not captured by age-based models, residual diagnostics relating model residuals to stand basal area and stem density were conducted and are presented in the supplementary material (Fig.S2-S5). All datasets and R scripts used in this study are publicly available at Zenodo^35^.

## Supplementary Information

Below is the link to the electronic supplementary material.

Supplementary Material 1

Supplementary Material 2

Supplementary Material 3

## Data Availability

The minimum dataset and R code required to reproduce all main results and figures in this study are publicly available at Zenodo (https://doi.org/10.5281/zenodo.18337681).
