# Supplementary material for: Mangrove growth and biomass dynamics along the mud-dominated coast of French Guiana
Source: Sci Rep. 2026 May 21;16:15869. doi: 10.1038/s41598-026-53756-1 (PMC13194718; doi:10.1038/s41598-026-53756-1)
Supplement: Supplementary file 3 — Supplementary Material 3 [file 41598_2026_53756_MOESM3_ESM.pdf]

Mangrove growth and biomass dynamics along the mud-dominated coast of French Guiana

Michael Kyei Agyekum [1,2*], Joao Marcelo Brazao Protazio[3], Adrien Staquet [4, 5]
Paul-Emile Augusseau[4, 5], Antoine Gardel[5], Antoine Mury[4,6], Edward J Anthony[7]
Christophe Proisy[4]

Supplementary methods

Stand age reconstruction and uncertainty classification

Stand age was reconstructed from sequential historical aerial and satellite imagery spanning 1940–2022. All available georeferenced imagery from the IGN *Remonter le temps* archive, SPOT (1986–present), and Landsat 5,7, and 8 was visually inspected to determine the first appearance of persistent woody canopy at each plot location. Canopy presence was defined as a continuous crown signal that remained visible in all subsequent images. The estimated establishment year corresponds to the earliest image in which persistent canopy was detected. Stand age was then calculated as the difference between the year of field measurement and the estimated establishment year.

Because the temporal resolution of available imagery varies across decades, age estimates carry different levels of uncertainty. Plots established after 1980 benefit from dense SPOT, Landsat, and IGN aerial coverage, resulting in effective acquisition intervals of approximately 3–5 years. These stands were therefore assigned an uncertainty of ±5 years. In contrast, plots established in 1980 or earlier rely on sparse mid-century aerial archives, with typical gaps of 8–12 years between usable images. These stands were assigned a broader uncertainty of ±10 years. These uncertainty classes reflect the temporal spacing of available imagery rather than assumptions about mangrove growth (Table S2).

Table S3. Fitted parameters of nonlinear growth models for stand diameter (DBH) and above-ground biomass (AGB) of *Avicennia germinans* and *Rhizophora* spp.

Model parameters were estimated from plot-level stand age observations using nonlinear least-squares regression. Parameters θ_1, θ_2, and θ_3 describe asymptotic magnitude, rate, and shape components, respectively, depending on the functional form of each model. For the Power model, parameters *a* (scale) and *b* (shape) are reported instead of θ_1–θ_3. All values shown are fitted coefficients underlying the growth curves presented in Figs. 2-5.

Species	Response	Model	θ_1 (Asymptote)	θ_2/a (Rate/Scale)	θ_3/b (Shape)
Avicennia germinans	DBH	Power	—	5.98	0.62
		Gompertz	88.12	2.09	0.04
		Logistic	78.77	5.59	0.08
		Monomolecular	145.62	0.93	0.01
	AGB	Power	—	63.88	0.33
		Gompertz	234.21	1.28	0.08
		Logistic	232.39	2.27	0.10
		Monomolecular	237.74	0.77	0.06
***Rhizophora* spp.**	DBH	Power	—	3.52	0.37
		Gompertz	13.6	4.91	0.28
		Logistic	13.29	31.44	0.50
		Monomolecular	14.03	1.30	0.14
	AGB	Power	—	0.02	1.22
		Gompertz	13.90	5.04	0.08
		Logistic	13.73	64.11	0.15
		Monomolecular	18.38	1.08	0.02

Residual diagnostics against stand structure

To assess whether variation in stand structure helps explain departures from the age-only growth models, we conducted a residual diagnostic for each fitted age–response relationship. Analyses were performed separately for diameter at breast height (DBH) and above-ground biomass (AGB), and separately for *Avicennia germinans* and *Rhizophora* spp.

For each taxon and response variable, residuals were calculated as the difference between the observed stand-level value (mean DBH in cm, or AGB in tonnes of dry matter per hectare) and the value predicted by the selected nonlinear age-only model at the observed stand age. A residual value close to zero indicates good agreement between the model prediction and the observation. Positive residuals indicate stands that are larger or more biomass-rich than expected based on age alone, whereas negative residuals indicate stands that are smaller or less biomass-rich than predicted.

We then examined whether these residuals were related to stand structural attributes that were available in the dataset but were not included as covariates in the growth models. Specifically, we considered stand basal area (Gtot, expressed in square metres per hectare) and stem density (trees per hectare).

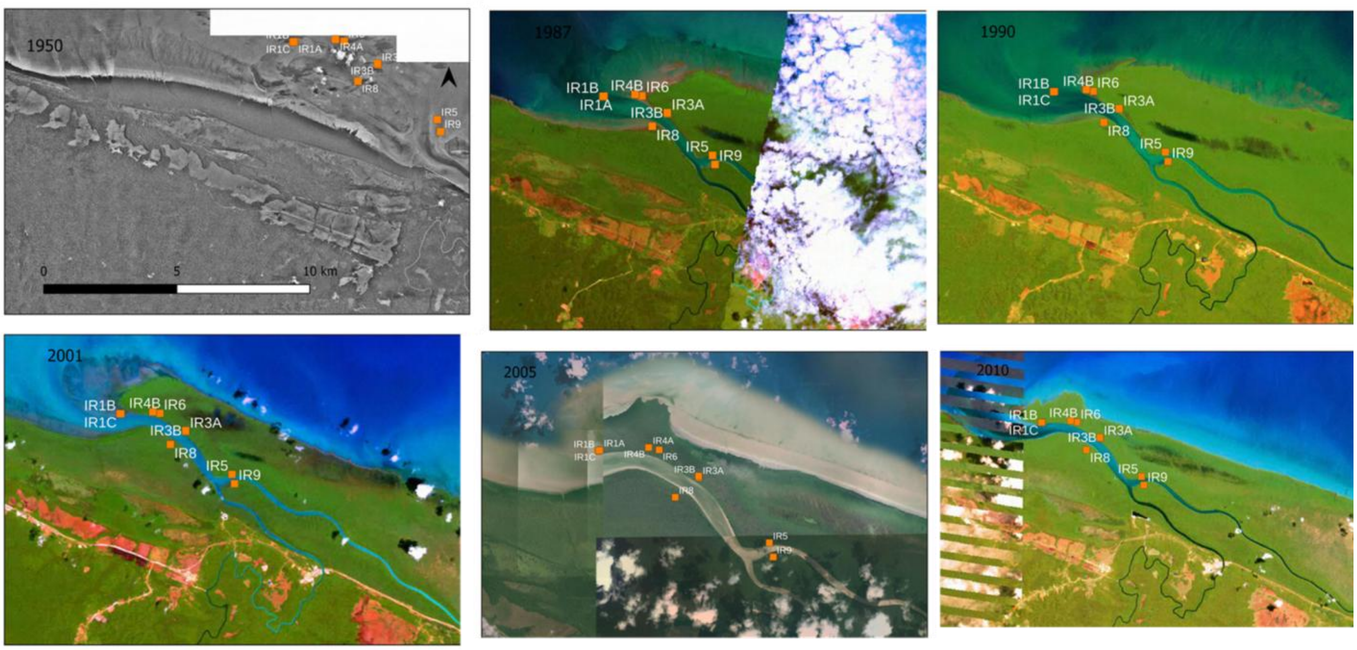

Fig. S1. Historical imagery used for stand-age reconstruction at Iracoubo, French Guiana. Georeferenced aerial photographs and satellite images from 1950, 1987, 1990, 2001, 2005, and 2010 illustrate progressive mudbank accretion and subsequent mangrove colonization along the Iracoubo coast. Orange squares indicate field plot locations used in this study. Stand establishment year was determined by visual interpretation of the earliest image in which persistent woody canopy appears at each plot location and remains visible in all subsequent images. Differences in colour balance, cloud cover, and striping reflect variations among sensors and acquisition conditions. These panels provide representative examples and do not show the full set of images inspected for stand-age reconstruction. The figure layout and annotations were produced in QGIS version 3.38.3 (QGIS Development Team; https://www.qgis.org) using historical imagery obtained from the IGN ‘Remonter le temps’ archive and SPOT and Landsat missions.

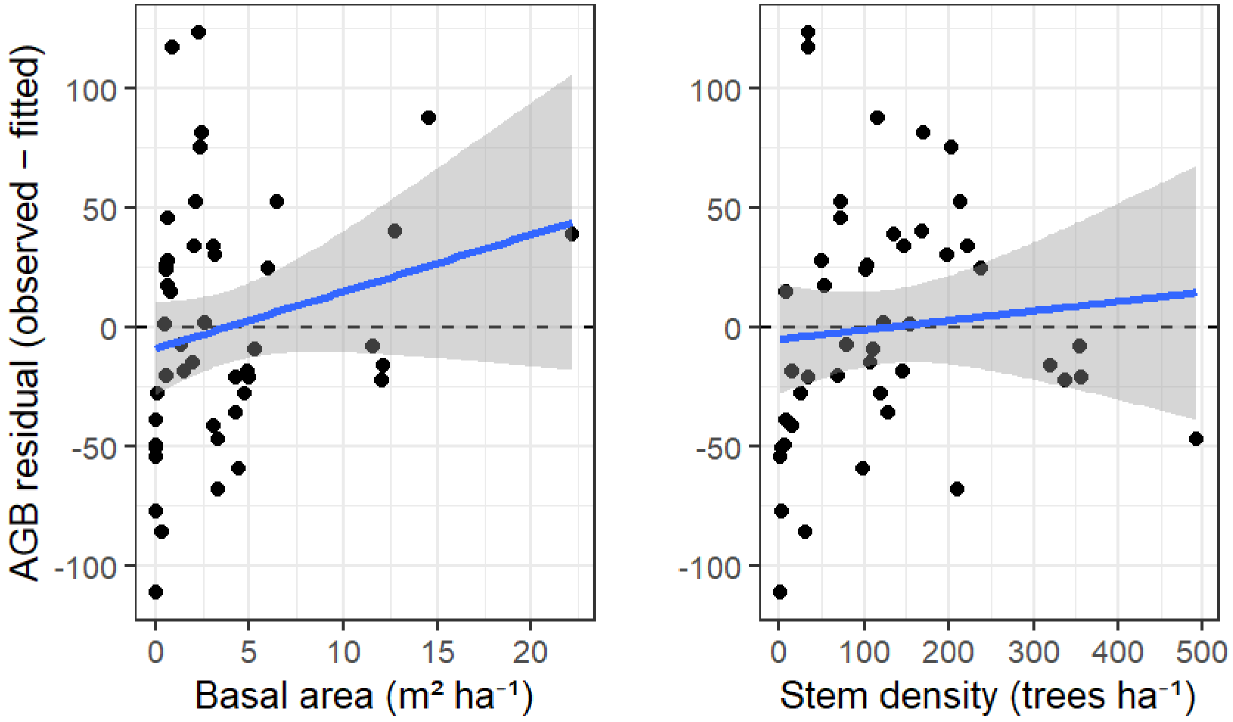

Fig. S2. Residual diagnostics for aboveground biomass (AGB) of *Avicennia germinans* in relation to stand structure.

Residuals from the age-only AGB growth model (observed minus fitted AGB, t DM ha^{-1}) are plotted against stand basal area (Gtot; m^2 ha^{-1}, left panel) and stem density (trees ha^{-1}, right panel). Points represent individual plots. The dashed horizontal line indicates zero residual. Solid lines show linear trends with 95% confidence intervals.

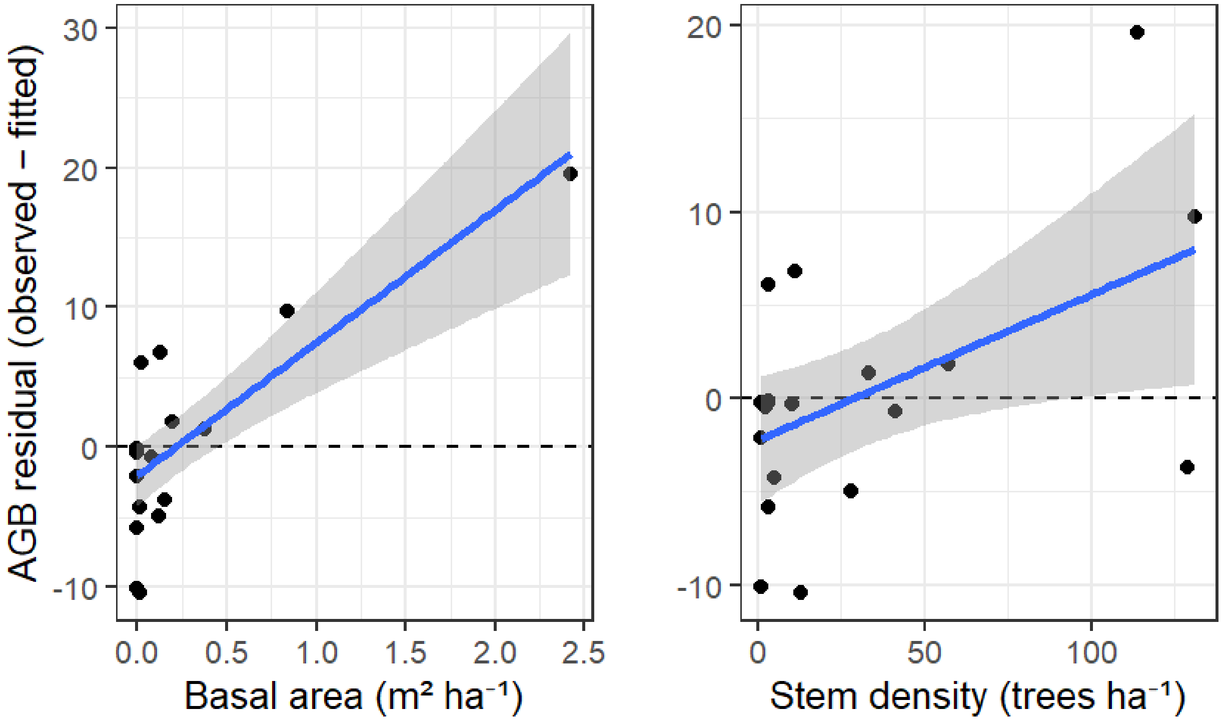

Fig. S3. Residual diagnostics for aboveground biomass (AGB) of *Rhizophora* spp. in relation to stand structure.

Residuals from the age-only AGB growth model (observed minus fitted AGB, t DM ha^{-1}) are shown as a function of stand basal area (Gtot; m^2 ha^{-1}, left panel) and stem density (trees ha^{-1}, right panel). Points represent individual plots. The dashed horizontal line indicates zero residual. Solid lines show linear trends with 95% confidence intervals.

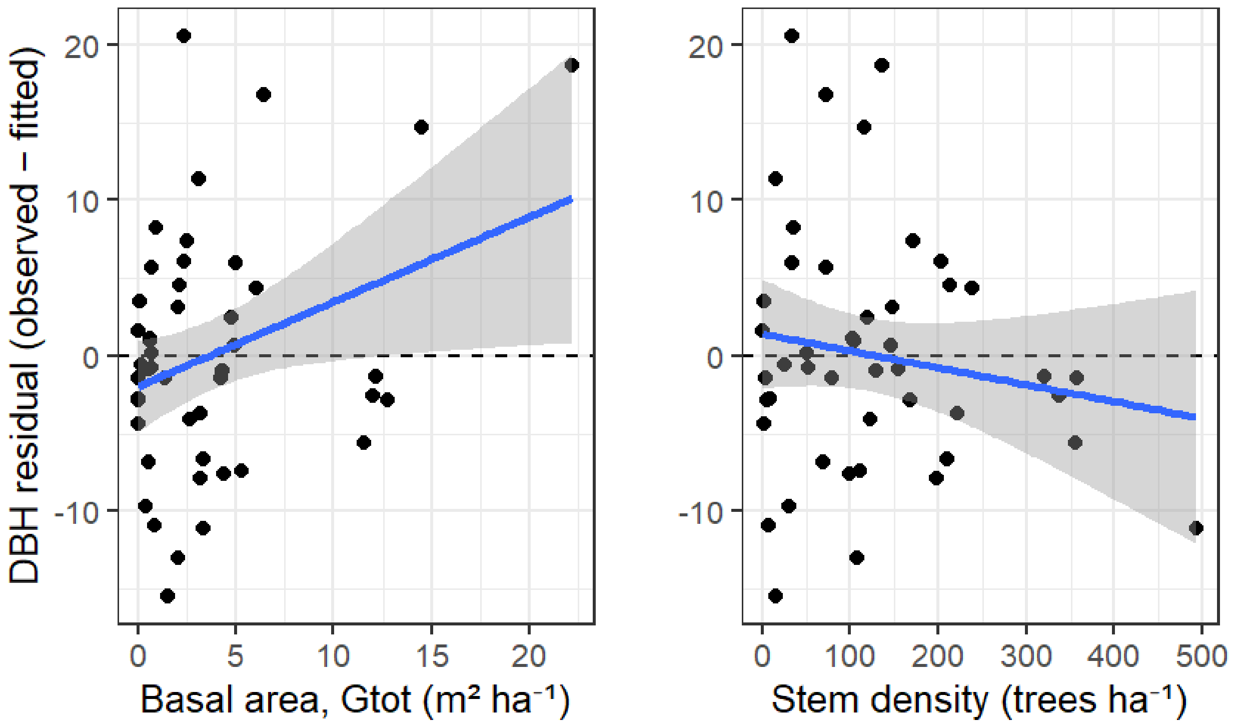

Fig. S4. Residual diagnostics for mean diameter at breast height (DBH) of *Avicennia germinans* in relation to stand structure.

Residuals from the age-only DBH growth model (observed minus fitted mean DBH, cm) are plotted against stand basal area (Gtot; $m^2\ ha^{-1}$, left panel) and stem density (trees ha^{-1}, right panel). Points represent individual plots. The dashed horizontal line indicates zero residual. Solid lines show linear trends with 95% confidence intervals.

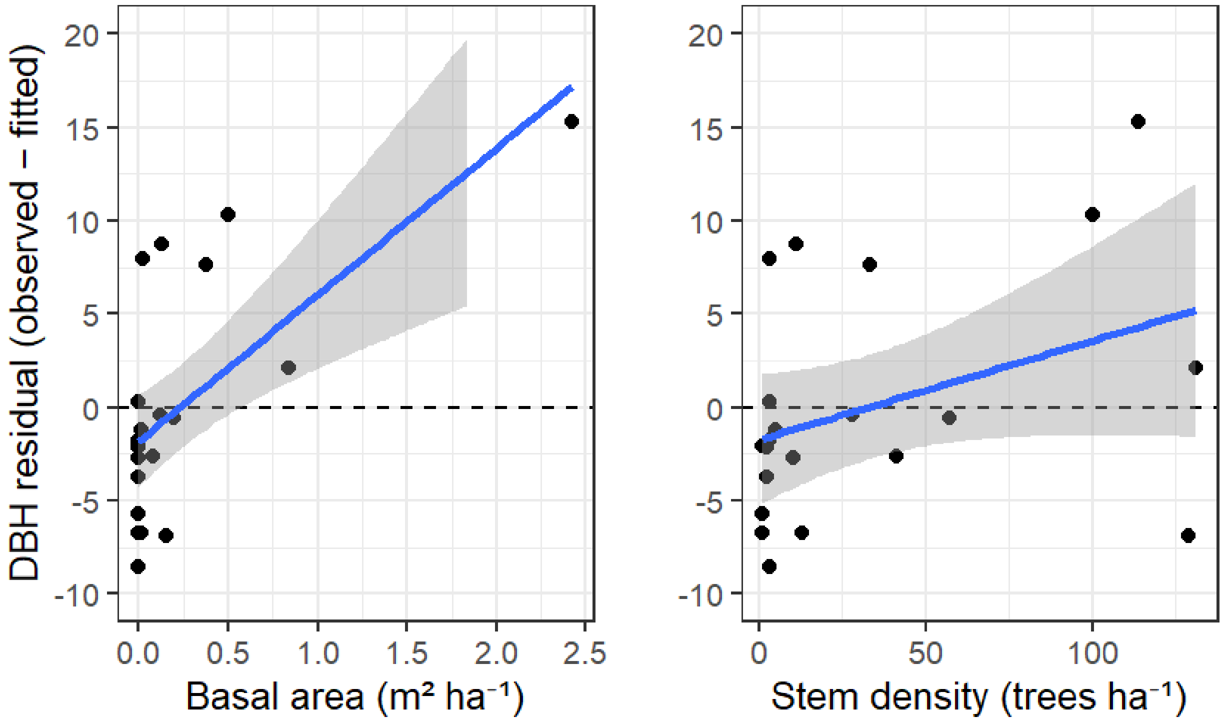

Fig. S5. Residual diagnostics for mean diameter at breast height (DBH) of *Rhizophora* spp. in relation to stand structure.

Residuals from the age-only DBH growth model (observed minus fitted mean DBH, cm) are displayed as a function of stand basal area (Gtot; m^2 ha^{-1}, left panel) and stem density (trees ha^{-1}, right panel). Points represent individual plots. The dashed horizontal line indicates zero residual. Solid lines show linear trends with 95% confidence intervals.
